# Big Defensins, a Diverse Family of Antimicrobial Peptides That Follows Different Patterns of Expression in Hemocytes of the Oyster *Crassostrea gigas*

**DOI:** 10.1371/journal.pone.0025594

**Published:** 2011-09-28

**Authors:** Rafael D. Rosa, Adrien Santini, Julie Fievet, Philippe Bulet, Delphine Destoumieux-Garzón, Evelyne Bachère

**Affiliations:** 1 IFREMER, CNRS, Université Montpellier 2, IRD, and Université Montpellier 1, UMR 5119 “Écologie des Systèmes Marins Côtiers”, Montpellier, France; 2 CNRS, Université Montpellier 2, IRD, and Université Montpellier 1, UMR 5119 “Écologie des Systèmes Marins Côtiers”, Montpellier, France; 3 CNRS AGIM FRE 3405, UJF-Grenoble 1-EPHE, La Tronche, France; 4 AGIM, Plateforme BioPark Archamps, Archamps, France; French National Centre for Scientific Research - Université de Toulouse, France

## Abstract

**Background:**

Big defensin is an antimicrobial peptide composed of a highly hydrophobic N-terminal region and a cationic C-terminal region containing six cysteine residues involved in three internal disulfide bridges. While big defensin sequences have been reported in various mollusk species, few studies have been devoted to their sequence diversity, gene organization and their expression in response to microbial infections.

**Findings:**

Using the high-throughput Digital Gene Expression approach, we have identified in *Crassostrea gigas* oysters several sequences coding for big defensins induced in response to a *Vibrio* infection. We showed that the oyster big defensin family is composed of three members (named *Cg*-BigDef1, *Cg*-BigDef2 and *Cg*-BigDef3) that are encoded by distinct genomic sequences. All *Cg*-BigDefs contain a hydrophobic N-terminal domain and a cationic C-terminal domain that resembles vertebrate β-defensins. Both domains are encoded by separate exons. We found that big defensins form a group predominantly present in mollusks and closer to vertebrate defensins than to invertebrate and fungi CSαβ-containing defensins. Moreover, we showed that *Cg-BigDef*s are expressed in oyster hemocytes only and follow different patterns of gene expression. While *Cg-BigDef3* is non-regulated, both *Cg-BigDef1* and *Cg-BigDef2* transcripts are strongly induced in response to bacterial challenge. Induction was dependent on pathogen associated molecular patterns but not damage-dependent. The inducibility of *Cg*-BigDef1 was confirmed by HPLC and mass spectrometry, since ions with a molecular mass compatible with mature *Cg*-BigDef1 (10.7 kDa) were present in immune-challenged oysters only. From our biochemical data, native *Cg*-BigDef1 would result from the elimination of a prepropeptide sequence and the cyclization of the resulting N-terminal glutamine residue into a pyroglutamic acid.

**Conclusions:**

We provide here the first report showing that big defensins form a family of antimicrobial peptides diverse not only in terms of sequences but also in terms of genomic organization and regulation of gene expression.

## Introduction

Big defensin is an antimicrobial peptide (AMP) initially characterized in a Chelicerate, the horseshoe crab *Tachypleus tridentatus*, whose immune system has been extensively studied [1]. The big defensin purified from horseshoe crab hemocytes is a 79 amino acid peptide with antimicrobial activities against both Gram-positive and Gram-negative bacteria and fungi [2]. The polypeptide is composed of a highly hydrophobic N-terminal region and a cationic C-terminal region containing six cysteine residues involved in three internal disulfide bridges. Interestingly, after experimental trypsin digestion at Arg-37 residue, the two generated peptides were reported to display distinct activities: the N-terminal peptide is more active against Gram-positive bacteria whereas the C-terminal cationic peptide is more active against Gram-negative bacteria. Besides, the native full-length big defensin displays significant LPS-binding properties whereas the two separated regions do not [2].

The solution structure of horseshoe crab big defensin showed that the N-terminal hydrophobic sequence adopts a unique globular conformation consisting in a parallel β-sheet and two α-helices [3]. Interestingly, the C-terminal region of the horseshoe crab big defensin forms a β-sheet structure folded by three disulfide bounds, which is similar to the three-stranded antiparallel β-sheet structure of the human β-defensin HBD-2 and HBD-3 [4], antimicrobial peptides active against both Gram-positive and Gram-negative bacteria [5]. Such a structure is different from that of invertebrate defensins, which consists of an α-helix linked to an antiparallel two-stranded β-sheet by 3 to 4 disulfide bridges [6]. This last structure is common to all mollusk defensins and was solved in both mussel and oyster defensins [7], [8].

In mollusks, big defensin-related sequences have been found in the hard clam [9] and oysters [10]. To date only one big defensin sequence has been cloned and recombinantly expressed in the clam *Venerupis philippinarum* [11] and in the bay scallop *Argopecten irradians* [12]. Like the native horseshoe crab big defensin [2], the scallop recombinant big defensin proved to be active against both Gram-positive and Gram-negative bacteria, and fungi.

Recent studies from our group and others have shown that AMPs from marine invertebrates have been the subject of intense diversifying selection [13], [14]. It has been shown that the different members of a given AMP family can be expressed in various cells and tissues. For instance, members of the defensin family are continuously expressed both in oyster mantle (*Cg*-Defm) [8] and hemocytes (*Cg*-Defh1 and *Cg*-Defh2) [15]. Various proline-rich AMPs, named *Cg*-Prps, are also expressed in oyster hemocytes [16]. Besides, a high level of sequence diversity has been found both at the transcript and genomic levels. Both *Cg*-Defs and *Cg*-Prps are multigenic families of AMPs [17]. Such diversity could greatly contribute to a broader antimicrobial response in oysters.

Interestingly, while big defensins have been largely studied in terms of primary structure and antimicrobial activities, all studies have been performed on one given sequence only. Surprisingly, little attention has been paid to their sequence diversity, phylogeny and gene organization and to their expression during the anti-infectious response. Herein, we have improved our knowledge of *C. gigas* antimicrobial response with the characterization of different members of the big defensin family.

Here, we have applied the high-throughput Digital Gene Expression (DGE) approach to identify genes potentially involved in oyster survival to *Vibrio* infections and have identified several EST sequences encoding big defensins named *Cg*-BigDef1, *Cg*-BigDef2 and *Cg*-BigDef3. We showed that they are encoded by different genomic sequences. Interestingly, whereas *Cg-BigDef1* and *Cg-BigDef2* were up-regulated in oyster hemocytes in response to a microbial challenge and to a pathogenic *Vibrio* infection, *Cg-BigDef3* was constitutively expressed. We provide here the first evidence of big defensin diversity in terms of sequence, gene regulation and genomic organization. Finally, from a phylogenetic analysis of big defensin sequences identified here and found in the GenBank database, we show that big defensins form a group predominant in mollusks and distinct from the vertebrate and invertebrate defensin-related families.

## Results

### Identification of big defensin sequences in oyster response to a *Vibrio* infection

Our group has developed a high-throughput Digital Gene Expression (DGE) approach in *Crassostrea gigas* to identify hemocyte genes potentially associated to the oyster capacity to survive virulent *Vibrio* infections [18]. Thus, three hemocyte DGE libraries were generated from oysters Surviving infection with Virulent (SVir) and aVirulent (SaVir) *Vibrio* strains and from Non-Infected (NInf) oysters. Library sequencing resulted in a total of 21,755 tag signatures differentially expressed (>2-fold change) which have been matched against the GigasDatabase [10]. From these analyses, a tag sequence (TAG1) mapped to two different EST sequences (GenBank: CU987401, AM853907) homologous to the horseshoe crab big defensin (**Table 1**). This tag displayed an occurrence of 111 in the NInf library and of 1866 in the SaVir DGE library and 1058 in the SVir one and it was 24-fold more abundant in the infected oyster libraries than in the non-infected one. Another tag (TAG2) matched a third big defensin sequence (GenBank: AM865249) with an occurrence of 108 in the NInf library and only 8 and 19 in SVir and SaVir libraries, respectively (**Table 1**).

**Table 1:** Assigned tag sequences specific to big defensins in oyster DGE libraries.

	Occurrences			GenBank	*Cg*-BigDef	E-value	
DGE tag sequence	NInf	SVir	SaVir	accession no.	form	(% identity)	GenBank description
TAG1 (CATGCAGAGATTACTG)	111	1058	1866	CU987401	*Cg*-BigDef1	3e-15 (49.4%)	Full = Big defensin [BDEF_TACTR: P80957]
				AM853907	*Cg*-BigDef2	2e-13 (50%)	Full = Big defensin [BDEF_TACTR: P80957]
TAG2 (CATGGATTAATCAGCC)	108	8	19	AM865249	*Cg*-BigDef3	8e-15 (54.3%)	Full = Big defensin [BDEF_TACTR: P80957]

NInf: non-infected DGE library; SVir: surviving virulent *Vibrio* DGE library; SaVir: surviving avirulent *Vibrio* DGE library.

The identified oyster EST sequences corresponded to full-length coding sequences (CDS) and displayed 49 to 54% identity to the big defensin of the horseshoe crab *T. tridentatus* (BDEF_TACTR, GenBank: P80957).

### Oyster big defensins represent a multi-domain defense peptide family composed of three members

The full-length cDNA sequences of three big defensins were cloned by PCR amplification from hemocyte RNA samples using specific primers and named *Cg*-BigDef1, *Cg*-BigDef2 and *Cg*-BigDef3. The *Cg*-BigDef1 cDNA sequence starts with a 5′ untranslated region (UTR) of 106 bp, followed by a CDS of 393 bp and a 3′-UTR of 242 bp containing a single polyadenylation signal (AATAAA), and finally a poly(A) tail. The CDS encodes a 130 amino acid prepropeptide (**Figure S1**). The two other sequences, *Cg*-BigDef2 and *Cg*-BigDef3, display variable 5′- and 3′-UTR sequences and a CDS of 372 bp encoding a 123 amino acid prepropeptide (**Figure S1**).

**Figure 1:**
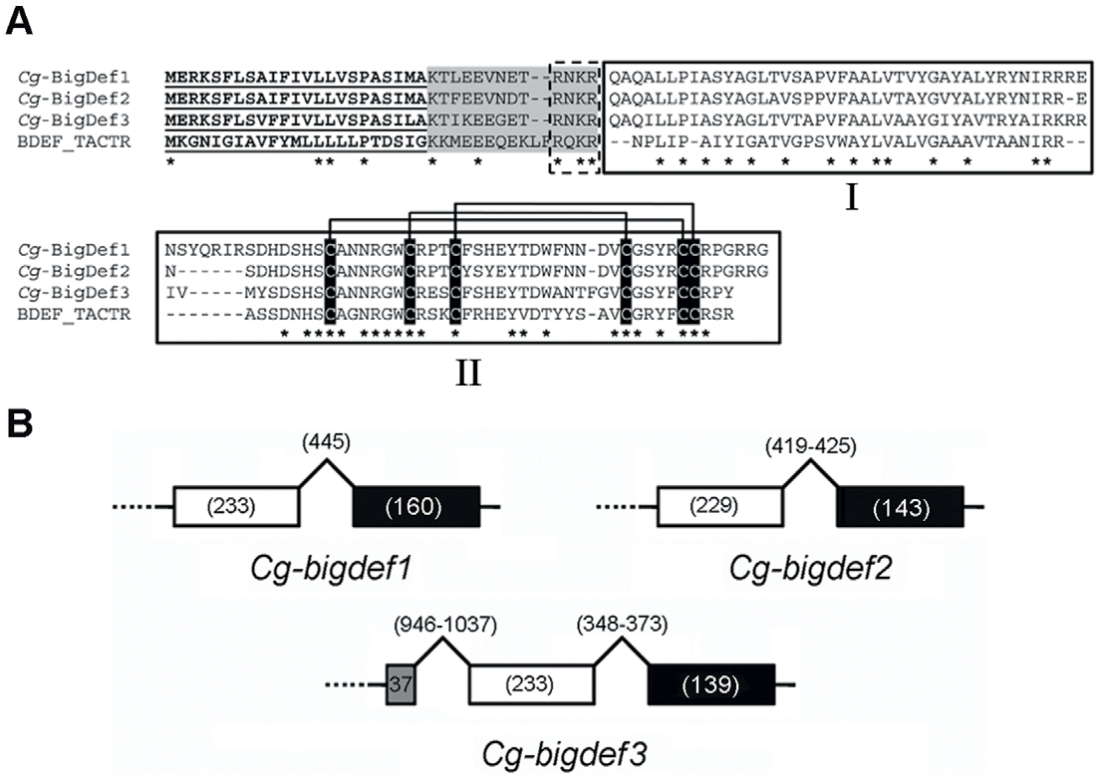
Oyster big defensin members are multi-domain defense polypeptides encoded by distinct genomic sequences. **a**: Amino acid sequence alignment of big defensins from *Crassostrea gigas* and *Tachypleus tridentatus* (BDEF_TACTR, GenBank: P80957). The predicted signal peptides are in bold and underlined. The putative propeptides and the conserved cysteine residues are shadowed with grey and black backgrounds, respectively. I: hydrophobic N-terminal domain; II: C-terminal cysteine-rich domain (β-defensin-like). The putative furin-like cleavage motif (RXKR) is indicated by a dotted rectangle. Asterisks (*) indicate identical amino acid residues. **b**: A not-to-scale representation of oyster big defensin genomic sequences (*Cg-bigdef1*, *Cg-bigdef2* and *Cg-bigdef3*). Black lines represent the untranslated regions of the exons. White boxes indicate the signal and the propeptide sequence, and the N-terminal domain of the mature peptide. Black boxes indicate the C-terminal domain of the mature peptides, and the grey box indicates a part of the 5′ untranslated region (37-bp) of the first exon found in *Cg-bigdef3*. Numbers indicate the length of exons and introns (in base pairs).

The deduced amino acid sequences of the three polypeptides start with a predicted 23-residue signal peptide, followed by a putative cationic propeptide region (p*I* range of 8.6 to 9.7) of 13 residues and a putative mature peptide of 94 residues for *Cg*-BigDef1 and 87 residues for *Cg*-BigDef2 and *Cg*-BigDef 3 (**Figure 1a**). Using the ProP method, a propeptidase cleavage site was identified at the end of the propeptide sequence. Indeed, its last four residues form a Arg-Asn-Lys-Arg motif, conserved in all *Cg*-BigDefs, that falls into the Arg-X-(Arg/Lys)-Arg target sequences for furin-like processing enzymes (proprotein convertase furin or dibasic-processing enzyme). Interestingly, we also identified a furin-like convertase in GigasDataBase (GenBank: FP004575, CU989616, AM862886) that presents about 60–80% amino acid similarity with furin-like prohormone convertases from the gastropods *Lymnaea stagnalis* and *Aplysia californica* [19], [20]. Altogether, this strongly supports the hypothesis of a maturation of the *Cg*-BigDefs precursors at the end of the 13-residue propeptide sequence. The resulting putative mature *Cg*-BigDefs consist in a highly hydrophobic N-terminal sequence and a cationic C-terminal sequence containing six cysteine residues at positions conserved in horseshoe crab big defensin and in vertebrate β-defensins (**Figure 1a**). Besides, putative mature big defensins are highly cationic with a calculated p*I* ranging from 8.6 to 9.2. *Cg*-BigDef1 shares 90% and 75% amino acid sequence identity with *Cg*-BigDef2 and *Cg*-BigDef3, respectively whereas *Cg*-BigDef2 and *Cg*-BigDef3 share 68% identity.

### Big defensins form a diverse family of oyster defense peptides

*In silico* analysis of GigasDataBase led to the discovery of different isoforms for each oyster big defensin: seven were found for *Cg*-BigDef1 (GenBank: JF703137 to JF703143), three for *Cg*-BigDef2 (GenBank: JF703144 to JF703146) and eight for *Cg*-BigDef3 (GenBank: JF703147 to JF703154). Among the isoforms identified for *Cg*-BigDef1 and *Cg*-BigDef3, five and four of them differed at the amino acid level through six non-synonymous nucleotide substitutions, respectively. In contrast, the three *Cg*-BigDef2 isoforms only presented synonymous nucleotide substitutions. The 3′ region, that codes the C-terminal domain of the mature peptide, is the most conserved region of the CDS with only few nucleotide substitutions. Besides, no *indel* events were observed in the isoforms of the different big defensins. Although many substitutions were found in the *Cg*-BigDefs, both signal peptide and propeptide cleavage sites remained conserved as well as the position of the six cysteine residues in the β-defensin-like domain.

### Oyster big defensins are encoded by distinct genomic sequences

The genomic organization of the three *Cg-*BigDef forms was investigated by PCR-based cloning and sequencing of the encoding regions of oyster genomic DNA (GenBank: JF703155 to JF703160). Five oysters have been considered for this partial gene characterization which revealed that each of the three oyster big defensins is encoded by a distinct genomic sequence. *Cg-bigdef1* and *Cg-bigdef2* genomic organization was similar, revealing the presence of two exons interrupted by a single intron. For these forms, the first exon covers part of the 5′-UTR, the signal peptide, the propeptide sequence and the N-terminal domain of the mature peptide. The second exon covers the cysteine-rich C-terminal (β-defensin-like) region and part of the 3′-UTR sequence (**Figure 1b**). In contrast, in *Cg-bigdef3*, additional intron and exon were observed upstream the first exon common to the *Cg-bigdef*s. The *Cg-bigdef3* additional exon covers part of the 5′-UTR found in the cDNA sequence (37-bp) (**Figure 1b**).

The length and nucleotide composition of the exons differ among the three big defensin genomic coding sequences. Besides, for each *Cg-bigdef*, the exon length appears conserved among individuals whereas the intron lengths are variable (**Figure 1b**).

### Big defensins cluster in a separated clade from other defensins families

The deduced amino acid sequences of *Cg*-BigDef1, -2 and -3 were compared with big defensin sequences from the horseshoe crab *T. tridentatus* (GenBank: P80957), the amphioxus *Branchiostoma belcheri tsingtauense* and *B. floridae* (GenBank: AAO18674, ADH03419), the scallop *A. irradians* (GenBank: ABC61319) and the clam *Venerupis* (*Ruditapes*) *philippinarum* (GenBank: ADM25826). Additionally, for this analysis, we identified several new translated EST sequences homologous to big defensins in different mollusk species in the GenBank database. These include sequences from the oyster *C. virginica* (BDEF_CRAVI: CV133156), the mussels *Mytilus galloprovincialis* (BDEF_MYTGA: FL490131), *M. californianus* (BDEF1_MYTCA: GE761911; BDEF2_MYTCA: GE759807) and *Hyriopsis cumingii* (BDEF_HYRCU: GW692819), the clam *V. philippinarum* (VpBD-EST: AM873974) and the abalone *Haliotis diversicolor* (BDEF_HALDS: GT870909).

**Figure 2:**
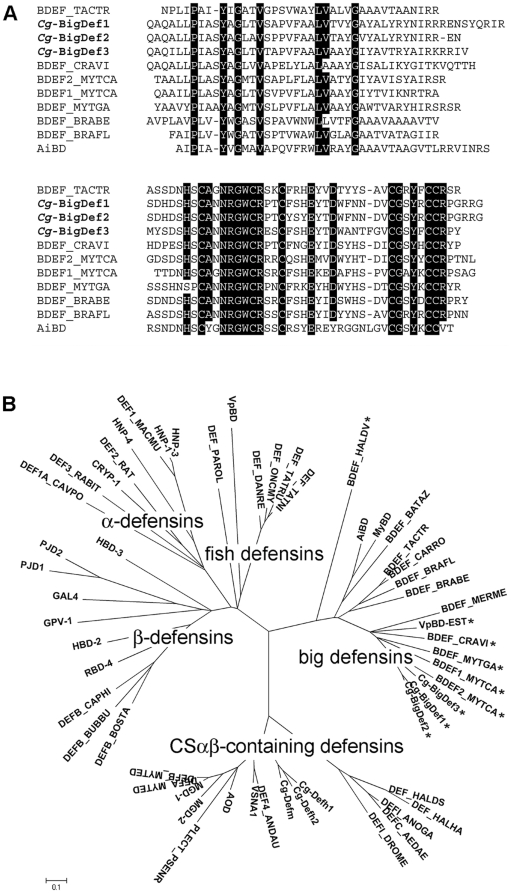
Big defensins form a group predominantly present in mollusk species and closer to vertebrate defensins. **a**: Multiple alignments of mature polypeptides of big defensins from bivalve mollusks, horseshoe crab and amphioxus. Identical amino acid residues are shaded with black backgrounds. **b**: Phylogenetic tree of different defensin groups, including big defensins, CSαβ-containing defensins, α-defensins, β-defensins and β-defensin-like peptides from fish. The tree was constructed using the Neighbour-Joining method in MEGA 4. Bootstrap sampling was reiterated 1,000 times. Sequences included in analyses of defensins were the following, where asterisks (*) indicate mollusk big defensin members newly identified in this study from GenBank: (i) big defensins: oysters *Crassostrea gigas* (*Cg*-BigDef1*: AEE92768, *Cg*-BigDef2*: AEE92775, *Cg*-BigDef3*: AEE92778) and *C. virginica* (BDEF_CRAVI*: CV133156), horseshoe crabs *Tachypleus tridentatus* (BDEF_TACTR: P80957) and *Carcinoscorpius rotundicauda* (BDEF_CARRO: CK086629), amphioxus *Branchiostoma belcheri tsingtauense* (BDEF_BRABE: AAO18674) and *B. floridae* (BDEF_BRAFL: ADH03419), scallops *Argopecten irradians* (AiBD: ABC61319) and *Mizuhopecten yessoensis* (MyBD: GH736001), clams *Venerupis* (*Ruditapes*) *philippinarum* (VpBD: ADM25826, VpBD-EST*: AM873974) and *Mercenaria mercenaria* (BDEF_MERME: GO915266), mussels *Mytilus galloprovincialis* (BDEF_MYTGA*: FL490131), *M. californianus* (BDEF1_MYTCA*: GE761911, BDEF2_MYTCA*: GE759807) and *Bathymodiolus azoricus* (BDEF_BATAZ: HM756150), abalone *Haliotis diversicolor* (BDEF_HALDV*: GT870909); (ii) CSαβ-containing defensins: oysters *C. gigas* (*Cg*-Defm: CAJ19280, *Cg*-Defh1: ABD66301, *Cg*-Defh2: ABD66302) and *C. virginica* (AOD: P85008), mussels *M. edulis* (DEFA_MYTED: P81610, DEFB_MYTED: P81611) and *M. galloprovincialis* (MGD-1: P80571, MGD-2: AAD52660), abalones *Haliotis discus discus* (DEF_HALDS: ACZ15982) and *H. discus hannai* (DEF_HALHA: ABF69125), scorpion *Androctonus australis* (DEF4_ANDAU: P56686), tick *Dermacentor variabilis* (VSNA1: AAO24323), fruit fly *Drosophila melanogaster* (DEFI_DROME: P36192), mosquitos *Anopheles gambiae* (DEFI_ANOGA: Q17027) and *Aedes aegypi* (DEFC_AEDAE: P81603), saprophytic fungus *Pseudoplectania nigrella* (PLECT_PSENR: Q53I06); (iii) α-defensins: rhesus monkey *Macaca mulatta* (DEF1_MACMU: P60030), house mouse *Mus musculus* (CRYP-1: NP_034161), Norway rat *Rattus norvegicus* (DEF2_RAT: Q62715), rabbit *Oryctolagus cuniculus* (DEF3_RABIT: P01376), domestic guinea pig *Cavia porcellus* (DEF1A_CAVPO: P11478), *Homo sapiens* (HNP-1: NP_004075, HNP-3: AAA35753, HNP-4: NP_001916); (iv) β-defensins: spiny lobster *Panulirus japonicus* (PJD1: ACM62357, PJD2: ACM62358), chicken *Gallus gallus* (GAL4: NP_001001610), wild turkey *Meleagris gallopavo* (GPV-1: AAG09213), *R. norvegicus* (RBD-4: NP_071989), water buffalo *Bubalus bubalis* (DEFB_BUBBU: ABI36600); cattle *Bos taurus* (DEFB_BOSTA: CAC15400), goat *Capra hircus* (DEFB_CAPHI: ABF71365), *H. sapiens* (HBD-2: NP_004933, HBD-3: NP_061131); (v) fish defensins: rainbow trout *Oncorhynchus mykiss* (DEF_ONCMY: ABR68250), zebrafish *Danio rerio* (DEF_DANRE: AM181358), fugu pufferfish *Takifugu rubripes* (DEF_TATRU: CAJ57646), spotted green pufferfish *Tetraodon nigroviridis* (DEF_TATNI: CAJ57644), Japanese flounder *Paralichthys olivaceus* (DEF_PAROL: ADA84138).

Amino acid sequence identity of *Cg*-BigDef1, -2 and -3 with the big defensin from horseshoe crab *T. tridentatus* was in the range of 48 to 53%, and of 47 to 55% with the amphioxus species, *B. belcheri tsingtauense* and *B. floridae*, as determined by ClustalW2 alignment (**Figure 2a**). Within mollusks, *Cg*-BigDefs were 55 to 67% identical to mussel sequences (*M. galloprovincialis* and *M. californianus*), 57% identical to an oyster sequence (*C. virginica*), 55 to 59% identical to EST-derived sequences from clams (*V. philippinarum* and *Mercenaria mercenaria*), 39 to 45% identical to scallop sequences (*A. irradians* and *Mizuhopecten yessoensis*), 41% identical to the deep sea hydrothermal vent mussel *Bathymodiolus azoricus*, and only 25 to 28% identical to the gastropod *H. diversicolor* sequence. However, less than 17% amino acid identity was observed between oyster big defensins and VpBD, recently reported as a big defensin in the clam *V. philippinarum* [12].

A phylogenetic tree was constructed with all big defensins mentioned above and defensin families from various species and phyla. In this tree, the defense peptides were split into five distinct groups: CSαβ-containing defensins from fungi and invertebrates, big defensins from invertebrates, as well as α-defensins, β-defensins and fish β-defensin-like peptides (**Figure 2b**). Oyster big defensins clustered together with other big defensin sequences, with exception of the clam VpBD sequence (GenBank: ADM25826) that showed no clear relationship with big defensins. Instead, this sequence clustered with β-defensin-like peptides from fish. Within the big defensin group, oyster big defensins appeared closely related to each other and with big defensins from *C. virginica*, *M. mercenaria*, mussels from the genus *Mytilus* and with a big defensin EST-derived sequence we identified in *V. philippinarum* (**Figure 2b**). Big defensins from horseshoe crabs, amphioxus and other mollusk species clustered in distinct groups. The phylogenetic tree also indicated that the big defensin cluster is closer to vertebrate α- and β-defensins than to invertebrate CSαβ-containing defensins.

### *Cg-BigDefs* are differentially modulated upon a bacterial challenge

**Figure 3:**
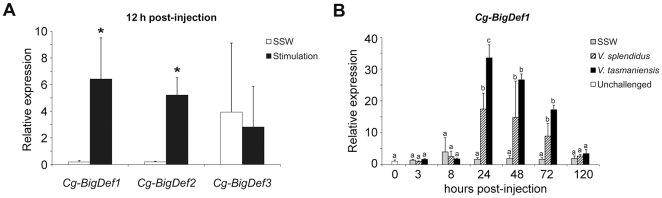
Relative expression of *Cg-BigDef* transcripts in oyster hemocytes by real-time quantitative PCR. **a**: Expression analysis of the three *Cg-BigDef* forms at 12 h post-stimulation with sterile sea water (white bars, SSW) and heat-killed bacteria (black bars, stimulation). Asterisks (*) indicate significant differences between conditions according to the Student's t-test (*p*<0.05). **b**: Time-course expression levels of transcripts of the inducible *Cg-BigDef1* at 0, 3, 8, 24, 48, 72 and 120 h after *Vibrio splendidus* LGP32 and *V. tasmaniensis* LMG 20012^T^ challenge or SSW injection. Data analysis was performed with the Pfaffl method. Significant differences (Student's t-test, *p*<0.05) are indicated by different lowercase letters (a, b, and c). The absence of significant difference is indicated by the use of identical lowercase letters.

The expression pattern of oyster big defensins was studied by quantitative PCR (qPCR) in response to a bacterial challenge. Oysters were injected with a mix of heat-killed Gram-positive and Gram-negative bacteria. A significant increase in *Cg-BigDef1* (*p*<0.025) and *Cg-BigDef2* (*p*<0.002) transcripts was observed in circulating hemocytes 12 h post-stimulation comparatively to a sterile sea water (SSW) control injection (**Figure 3a**). In contrast, no changes in *Cg-BigDef3* transcript abundance were seen following injection of bacteria or SSW. However, for *Cg-BigDef3*, an unusual high variability in transcript abundance was observed between the oyster hemocyte pools in all experimental conditions, independently of the bacterial or SSW challenge (**Figure 3a**).

The expression of *Cg-BigDef1* and *Cg-BigDef2*, which was modulated upon injection of heat-killed bacteria, was further analyzed in response to oyster experimental infections (intramuscular injection) with the virulent *Vibrio splendidus* LGP32 [21] and the avirulent *V. tasmaniensis* LMG 20012^T^ strains, belonging to the *V. splendidus* polyphyletic group [22]. In a control experiment, the injection of SSW (control) did not significantly modify the expression of *Cg-BigDef1* and *Cg-BigDef2* compared to unchallenged oysters. Conversely, the transcript abundance of *Cg-BigDef1* and *Cg-BigDef2* significantly increased after a *Vibrio* injection 8 h post-injection, reaching a peak at 24 h and slowly decreasing after 48, 72 and 120 h (**Figure 3b**). At the peak of expression (24 h), *Cg-BigDef1* gene expression was 16-fold (*p*<0.004) and 31-fold (*p*<0.0001) higher for animals infected with the virulent and avirulent *Vibrio* strains, respectively, than in unchallenged oysters. Only at this time post-injection, a significant difference in gene expression (*p*<0.011) was observed according to infection with the virulent or the avirulent *Vibrio* (**Figure 3b**). In the same conditions, *Cg-BigDef2* gene expression was similar to that of *Cg-BigDef1* (data not shown). This corroborated the DGE data (see above). A great variability in transcript abundance was observed for both *Cg-BigDef1* and *Cg-BigDef2*, particularly upon *V. splendidus* LGP32 infection.

### *Cg-BigDef* expression is restricted to circulating and tissue-infiltrating hemocytes

**Figure 4:**
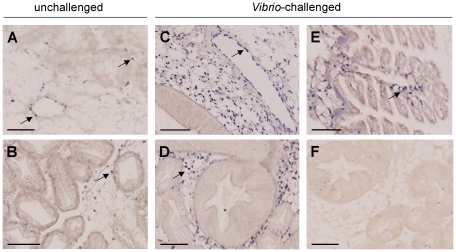
Localization of *Cg-BigDef* mRNA expression in unchallenged and *Vibrio*-infected oyster tissues by *in situ* hybridization (ISH). Histological sections of *C. gigas* were analyzed by ISH using *Cg-BigDef* -DIG-UTP antisense (**a-e**) and sense (**f**) riboprobes. In unchallenged oysters, *Cg-BigDef1* (**a**) and *Cg-BigDef3* (**b**) labeling appeared in hemocytes (arrows) located in blood vessels and infiltrating oyster tissues. In *Vibrio*-challenged oysters, strong hybridization signals were detected with *Cg-BigDef1* antisense riboprobe in hemocytes located in blood vessel (**c**) and invading massively connective tissues of the oyster organs such as the digestive gland (**d**) and the gills (**e**). Control sections with the sense *Cg-BigDef1* riboprobe were devoid of labeling (**f**). (Scale bars: 100 µm).

The localization of gene expression of the inducible *Cg*-*BigDef1* and constitutive *Cg*-*BigDef3* in unchallenged and *V. splendidus* LGP32-infected oysters was further studied by ISH using specific DIG-labeled riboprobes. In unchallenged oysters, the gene expression of *Cg*-*BigDef1* and *-3* forms was clearly restricted to hemocytes, both circulating, as seen in blood vessels and sinus, and infiltrating tissues. *Cg-BigDef* transcripts were detected in hemocytes infiltrating different oyster organs, such as the gills, the gonads and the digestive gland (**Figures 4a and 4b**). Consistent with our qPCR data, upon bacterial challenge of oysters, *Cg*-*BigDef3* expression was not modulated whereas *Cg*-*BigDef1* was strongly stimulated. Indeed, a striking increase in the number of *Cg*-*BigDef1* positive cells was observed in hemolymph vessels and connective tissues (**Figures 4c, 4d and 4e**) of the oysters at 24 h after *V. splendidus* LGP32 injection compared to non-injected animals. No signals were observed on histological sections hybridized with sense riboprobes (**Figure 4f**).

### Detection of *Cg*-BigDef1 in oysters subjected to bacterial challenge

The native *Cg*-BigDefs were finally investigated at the peptide level by RP-HPLC and mass spectrometry in oysters subjected to a bacterial challenge. Upon challenge, hemocytes expressing big defensins were seen highly infiltrating tissues including the gills (**Figure 4e**). We therefore compared the peptide profile of gill acid extracts at different times (12, 24 and 48 h) after an injection of heat-killed bacteria. Acid extracts were subjected to RP-HPLC, all RP-HPLC fractions being collected and subjected to MALDI-TOF MS.

**Figure 5:**
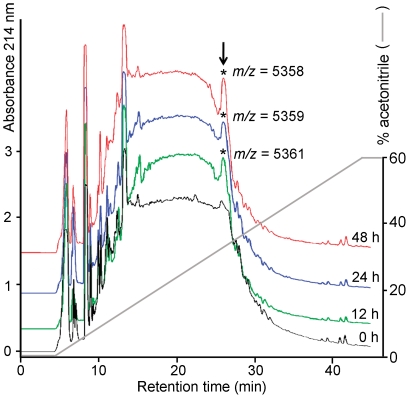
Reversed-phase HPLC of gill acid extracts from unchallenged (black line) and immune-challenged oysters analyzed 12 h (green line), 24 h (blue line), and 48 h (red line) after an injection of heat-killed bacteria. Elution was performed with a linear gradient of 0-60% of acetonitrile in acidified water over 40 minutes at a flow rate of 0.6 ml/min (grey line). Absorbance was monitored at 214 nm. One fraction eluted with 34% acetonitrile (arrow) showed an increased absorbance over the time course. Ions compatible with the mass of a double-charged *Cg*-BigDef1 (asterisks) were identified by MALDI-TOF MS in this fraction only. They were found in challenged oysters at 12, 24 and 48 h (see *m/z* values) but not in unchallenged oysters.

Because the actual state of mature *Cg-*BigDefs was unknown, we performed a blind screen of all the RP-HPLC fractions for the presence of ions corresponding to putative maturation states of all the *Cg-*BigDef forms and isoforms characterized in this study. Potential maturations included not only the oxidation of the disulfide bridges, but also the presence a pyroglutamic acid at position 1 (instead of the glutamine) and the elimination of the C-terminal glycine leading to a C-terminal amidation. Only one fraction displayed ions with *m/z* values corresponding to double charged ([M+2H]^2+^) *Cg-*BigDefs at all times after challenge. These ions were absent from the unchallenged oysters. Interestingly, this fraction, which eluted at 34% acetonitrile, corresponded to one peak that was almost absent from unchallenged oysters but significantly increased in absorbance after bacterial challenge (**Figure 5**). Ions of interest were detected at 12 h (*m/z* 5361), 24 h (*m/z* 5359) and 48 h (*m/z* 5358). Such masses correspond to the putative double charged ions of three isoforms of *Cg*-BigDef1, namely *Cg*-BigDef1-4 (GenBank: JF703141, [M+2H]^2+^ at calculated *m/z* 5361), *Cg*-BigDef1-5 and *Cg*-BigDef1-6 (GenBank: JF703142 and JF703143, [M+2H]^2+^ at calculated *m/z* 5360.5) assuming the following post-translation modifications: (i) elimination of the 13-residue propeptide region, (ii) cysteine bridge oxidization (loss of 6 Da), and (iii) cyclization of the N-terminal glutamine into a pyroglutamic acid (loss of 18 Da).

## Discussion

Here, we showed for the first time that oyster big defensins, *Cg*-BigDefs, form a family of antimicrobial peptides diverse in terms of sequences, genomic organization and gene expression regulation. By cDNA cloning we showed that *Cg*-BigDefs are composed of a 23-residue signal peptide followed by two cationic regions, namely a 13-residue propeptide sequence (calculated p*I* 8.6 to 9.7), and a C-terminal 87- to 94-residue sequence corresponding to the mature peptide (calculated p*I* 8.6 to 9.2). These two regions are separated by a propeptidase cleavage site Arg-X-(Arg/Lys)-Arg, a conserved motif for processing by furin, which in oyster hemocytes could be encoded by three EST sequences (GenBank: FP004575, CU989616, AM862886). The structure of oyster big defensin precursors differs from that of oyster defensins (*Cg*-Defs). Indeed, *Cg-*Defs derive from a precursor peptide composed of a signal peptide and a C-terminal cationic sequence, but lack the propeptide sequence. To date, nothing is known on the function of big defensin propeptide sequence. Because of its cationic charge, the propeptide in big defensins is unlikely used for trans inactivation of the mature AMP, as described for human α-defensins [23]. One can speculate that, as shown for the pro-domain of insect attacin C [24], the oyster big defensin propeptide could rather generate a second AMP. Indeed, it is not only cationic but also predicted to fold into an α-helical conformation by PSIPRED v3.0 software (data not shown), two well-known properties for membrane disrupting AMPs [25].

We showed here that the oyster big defensin family is composed of three members, encoded by three distinct genomic sequences. Besides, additional sequence diversity was observed (isoforms) for each member. Our data suggest that the big defensin molecular diversity is generated by single point mutation or the presence of different polymorphic alleles within the population, rather than by alternative splicing, which is a common way to generate sequence diversity [26]. We showed here that the genomic sequences encoding the three *Cg*-BigDefs differ in terms of structure (**Figure 1b**). Therefore, the big defensin may be a multigenic family of diversified members, as known to occur for other defense peptides in animal species and in particular in oyster. Indeed, to date, our group has evidenced that oyster defensins were a multigenic family generating high molecular diversity of sequences expressed from hemocytes (*Cg*-Defhs) and from mantle (*Cg*-Defm) [13]. More than 30 different defensin isoforms were sequenced and about 13 copies of *Cg*-Defhs and *Cg*-Defm encoding genes were estimated in solely three animals analyzed. In the human genome, the β-defensin genes are organized in a cluster of a minimum of seven β-defensins and this cluster can be found from 2 to 7 or 12 copies according to individuals [27]. It is noteworthy that the polymorphism of immune genes including antimicrobials may result in variable expression level, potentially causative of variability in pathogen or disease susceptibility [28], [29], [30]. The unique diversity of oyster *Cg*-BigDefs has not been reported previously in other species. Indeed, to date, reports on big defensins have mainly focused on one sequence only [2], [12].

Another major result from this study is that members of the *Cg*-BigDef family follow different gene regulation patterns in response to a bacterial challenge. The inducibility of *Cg-BigDef1* and *-2* was evidenced both by DGE approach and quantitative PCR on hemocytes from bacteria-challenged and *Vibrio*-infected oysters. Similarly, the single big defensin characterized in the bay scallop *A. irradians* was highly induced in circulating hemocytes after *V. anguillarum* challenge [12]. *Cg-BigDef1* and *-2* are the first AMPs found to be strongly induced in oyster hemocytes apart from the bactericidal/permeability-increasing protein, *Cg*-BPI [31]. Since no increase of *Cg*-*BigDef* transcript levels was evidenced following tissue injury (injection of sea water), the expression of the *Cg-BigDef1* and *-2* is likely induced by PAMPs (Pathogen-Associated Molecular Patterns). Interestingly, unlike *Cg-BigDef1* and *-2*, *Cg*-*BigDef3* gene expression seems to be constitutive and non-regulated in oyster hemocytes. This is similar to the regulation of oyster defensins (*Cg-*Defs) gene expression [15]. Future characterization of promoter regions of the three *Cg-bigdef* genomic sequences should help understanding their different expression patterns. In this study we also observed an unexplained individual variability of expression of *Cg-BigDefs* (mainly for *Cg-BigDef3*), which was not observed for *Cg-*Defs [15], and will require further attention.

Noteworthy, the differential regulation of *Cg-BigDefs* was observed in one same tissue, the hemocytes, as shown by *in situ* hybridization. Indeed, 24 h after *Vibrio* injection, only *Cg-BigDef1* transcripts were seen to be induced in hemocytes as evidenced by the great increase in hemocyte labeling intensity and number of positive cells in tissues, compared to unchallenged oysters (**Figure 4**). No other tissues than hemocytes were found to express *Cg-BigDefs*. This contrasts with the *Cg*-BPI which is not only expressed in hemocytes but also in epithelia, where its expression is not regulated in response to a microbial challenge [31]. Interestingly, in the hard clam *M. mercenaria*, transcript levels of a big defensin homolog have been reported to increase in the mantle and gills after challenge with the protistan parasite QPX (Quahog Parasite Unknown) [9], but probably following massive hemocyte infiltration of these tissues.

To our knowledge, this is the first report in a mollusk of an AMP family whose members are differentially regulated in hemocytes upon bacterial challenge. Like *Cg*-BigDefs, human β-defensins follow different regulation patterns in the intestinal epithelium. Indeed, while *HBD-1* is constitutively expressed, *HBD-*2 is regulated upon bacterial infection [32]. Similar to the vertebrate intestinal tract, oyster tissues are densely populated by diverse microbial communities dominated by *Vibrio* species [33]. Thus, tight mechanisms of regulation must be established in the antimicrobial defenses for maintaining homeostasis and controlling infections [34]. Big defensins have a broad spectrum of antimicrobial activities that affects *Vibrio* species [2], [12], which are common bacterial pathogens for oysters [21], [35]. Therefore, we can assume that AMPs constitutively expressed as defensins and *Cg*-BigDef3 may represent a watchful waiting line of defense whereas AMPs transcriptionally regulated such as *Cg*-BigDef1 and -2 may intervene upon microbial invasion.

Our HPLC and MS data strongly suggested that the mature form of native *Cg*-BigDef1 results from cleavage of both the signal peptide and the propeptide sequence, as well as the cyclization of the resulting N-terminal glutamine residue into a pyroglutamic acid. This last modification, commonly reported in bioactive peptides/polypeptides including series of AMPs, may contribute to the stabilization of the mature peptide and resistance to proteolysis [36]. That ions with a *m/z* value compatible with *Cg*-BigDef1 were only found in challenged oysters is consistent with the transcriptional regulation of *Cg-BigDef1*. In our acid extracts of oyster tissues, we could not observe any ion compatible with further cleavage of *Cg*-BigDef1 in two domains, namely the N-terminal hydrophobic domain and the C-terminal cationic domain. Altogether, this strongly suggests that mature *Cg*-BigDef1 is composed by both domains, a structure which was shown to provide wide antimicrobial activities and multifunctional properties to the horseshoe crab big defensin [2]. Interestingly, the C-terminal domain of big defensins present six cysteine residues whose arrangement is similar to those observed in vertebrate β-defensins [3], suggesting a phylogenetic relationship between β-defensin and big defensin peptides. Importantly, we show here for the first time that the hydrophobic N-terminal region and the C-terminal β-defensin-like domain are located in separated exons, a genomic architecture that has also been reported in multi-domain AMPs from crustaceans [37].

Finally, we showed that big defensins cluster in a separate clade distinct from other defensin families and closer to vertebrate defensins than to CSαβ-containing defensins. This was concluded from the phylogenetic analysis of defensin sequences from a broad variety of species. All big defensins fell into one group. Only the clam big defensin VpBD [11], which does not display the conserved propeptide sequence observed in the big defensins, clustered with β-defensin-like peptides from fish (**Figure 2b**). However, in this clam species as well as in other bivalve mollusks including scallops and mussels we found several big defensins that properly clustered with oyster big defensins. Importantly, from our phylogenetic analysis, the big defensin family appears to be predominantly represented in mollusk species.

### Conclusions

We show that big defensins represent in oyster a diverse multi-domain defense peptide family whose members are encoded by distinct genomic sequences and differentially regulated in hemocytes after bacterial challenge. These findings will help understanding the relationship and the respective implication of the constitutive and inducible antimicrobials in the oyster antimicrobial defense. In particular, attention must be paid on the diversity and variability of big defensin expression and their correlation with oyster survival capacities to pathogenic *Vibrio* infections.

## Materials and Methods

### Identification of big defensin homologues in oyster hemocytes by Digital Gene Expression (DGE) tag profiling

*Crassostrea gigas* DGE libraries were constructed with hemocyte mRNA precursors from non-infected and *Vibrio*-challenged oysters ([18], Rosa *et al*., in preparation). Briefly, a first DGE library (SVir DGE library) was constructed from pooled RNA samples of oysters Surviving infections with Virulent *V. splendidus* LGP32 [21] and *V. aestuarianus* LPi 02/41 [35], and a second library (SaVir DGE library) from individuals injected with the aVirulent *V. splendidus-*related strain *V. tasmaniensis* LMG 20012^T^ [22]. Hemocytes from Non-Infected animals were used for generating a control library (NInf DGE library). For each library, 7 µg of total RNA were used for sequence tag preparation using the Illumina's Digital Gene Expression Tag Profiling Kit according to the manufacturer's protocol (version 2.1B). Data from oyster DGE libraries were analyzed with BIOTAG software (Skuld-Tech, Montpellier, France) for tag detection, tag counting and for assessing DGE library quality, as described by Piquemal *et al*. [38]. About 14 millions of tags have been sequenced and 57,300 different tags have been identified, according to a level of sensibility established at 1 copy of mRNA per cell (1 copy for 300,000 molecules of mRNA). Obtained DGE tag signatures were matched against 29,745 unique transcribed sequences (7,940 contigs and 21,805 singletons) from different tissues of *C. gigas* (GigasDataBase; http://www.sigenae.org/aquafirst/). For tag to gene mapping, the virtual tags were extracted from all contigs and singletons. Only the full-length tags with 100% sequence identity with oyster expressed sequence tags (EST) were assigned. EST-matched sequences from DGE libraries were analyzed for similarities using BLASTX at the National Center for Biotechnology Information (NCBI; http://www.ncbi.nlm.nih.gov).

### Molecular cloning of oyster big defensins

Three distinct EST sequences homologous to the big defensin antimicrobial peptide from *T. tridentatus* (GenBank: P80957) were identified in *C. gigas* cDNA libraries. Specific primers were then designed to confirm the nucleotide authenticity of each big defensin form identified in oysters (**Table 2**). Total RNA was extracted from oyster hemocytes using TRIzol reagent (Invitrogen), treated with DNase I (Invitrogen) to eliminate contaminating genomic DNA and precipitated with 3 M sodium acetate. Following heat denaturation (70°C for 5 min), reverse transcription was performed using 1 µg of purified total RNA with 50 ng/µl oligo(dT)_12–18_ in a 20 µl reaction volume containing 1 mM dNTPs, 1 unit/µl of RNaseOUT Ribonuclease and 200 units/µl M-MLV reverse transcriptase in reverse transcriptase buffer according to the manufacturer's instructions (Invitrogen). PCR reactions were conducted in a 25-µl reaction volume using 1 µl of synthesized complementary DNA (cDNA) as template. PCR conditions were as follows: 30 cycles of 94°C for 1 min, 56°C for 1 min, 72°C for 1 min and a final elongation step of 72°C for 10 min. The amplification products were cloned into a pCR®-Blunt II-TOPO® cloning vector using a Zero Blunt® TOPO® PCR cloning kit (Invitrogen). The positive recombinant clones were identified by colony PCR and were sequenced in both directions.

**Table 2:** Nucleotide sequence of primers used in this study.

Primer name	Forward primer (5′−3′)	Reverse primer (5′−3′)
Primers for cDNA amplification		
*Cg-bigdef1* and *-2*	TGTTAACGTATAGGACTATC	GTAATCTCTGCATACATAGT
*Cg-bigdef3*	GTTAGTGTGCTGTGGCCAGAC	GGCTGATTAATCCATGCAAGC
Primers for genomic amplification		
*Cg-bigdef1g* and *-2g*	ACGTATAGGACTATCATGGAG	CGTGGCTCAGTAATCTCTGC
*Cg-bigdef3g1*	GGAGAACTGTTAGTGTGCTG	CCTGCATACGATGCTATGGG
*Cg-bigdef3g2*	AGAAGAAGGTGAGACGAG	TGGCTGATTAATCCATGCAAG
Primers for quantitative real-time PCR		
*Cg-rpl40qt*	AATCTTGCACCGTCATGCAG	AATCAATCTCTGCTGATCTGG
*Cg-bigdef1qt*	TTCGCCTGCTTCCATAATGG	GTCATGGTCACTCCTTATTC
*Cg-bigdef2qt*	TTCGCCTGCTTCCATAATGG	AATGACTGTCATGGTCAGAA
*Cg-bigdef3qt*	AGAAGAAGGTGAGACGAG	TGATCCGCACACACCAAACG
Primers for *in situ* hybridization		
*Cg-bigdef1ish*	TGTTAACGTATAGGACTATC	GTAATCTCTGCATACATAGT
*Cg-bigdef3ish*	AGAAGAAGGTGAGACGAG	GACGTGGCTGATTAATCC

### Sequence analysis

The obtained cDNA sequences were used in a directed search for novel variants in the *C. gigas* EST database. In addition, homologous sequences from other mollusks and unrelated species were also identified by screening of EST sequences available in GenBank. Homology searches were performed using BLAST at NCBI. The prediction of signal peptides and other post-translational processing were carried out using ProP method [39]. The deduced amino sequences were aligned by using the ClustalW2 Multiple Alignment program (ClustalW2; http://www.ebi.ac.uk/Tools/msa/clustalw2/). The phylogenetic analysis based on the deduced amino acid sequences of big defensins and other defensin families was performed using the Neighbour-Joining method within the software MEGA version 4.0 [40]. Bootstrap sampling was reiterated 1,000 times.

### Partial gene characterization

Genomic DNA (gDNA) was extracted from gills of five individual oysters using a standard phenol-chloroform extraction method followed by a treatment with RNase A (Invitrogen) and precipitation with 3 M sodium acetate. Genomic sequences for each oyster big defensin form were obtained by PCR amplification using gene-specific primers whose design was based on the cDNA sequences (**Table 2**). Amplifications were conducted in a final volume of 25 µl using 30 ng of gDNA under the following conditions: 10 min at 94°C, then 30 cycles at 94°C for 1 min, 56°C for 1 min, 72°C for 3 min and a final elongation step of 72°C for 10 min. PCR products were analyzed by electrophoresis on 1.5% (w/v) agarose gel and cloned and sequenced as described above.

### Oyster immune challenge and tissue collection

Adult 2 year-old *C. gigas* oysters were purchased from a local oyster farm in Mèze (Gulf of Lion, France) and kept in aquaria containing filtered sea water at 15°C at the Aquaculture Experimental Platform of IFREMER, Palavas-Les-Flots. The animals were fed twice with a mixture of live microalgae (*Nannochloropsis oculata*: 2.5×10^5^ cells/ml; *Tetraselmis suecica*: 2.5×10^2^ cells/ml). Bacterial challenges were performed as previously described by injecting bacteria into the oyster adductor muscle [41]. In a first stimulation assay, oysters were injected with 5×10^8^ CFU (Colony Forming Unit)/animal of heat-killed (100°C, 10^5^ Pa, 20 min) bacteria mixture (*Micrococcus luteus*, *V. splendidus* and *V. anguillarum*) under 100 µl sterile sea water (SSW). Injection of SSW (100 µl) was used as control. Hemolymph was collected 12 h post-stimulation from the oyster posterior adductor muscle sinus using a 2 ml syringe equipped with a 23G-needle. Hemocytes were obtained by centrifugation (15 min, 1,000 ×g, 4°C) of 3 hemolymph pools of 10 animals and directly processed for RNA extraction. Besides, gills from unchallenged and bacteria-challenged oysters at 12, 24 and 48 h were harvested by dissection and immediately frozen and conserved in liquid nitrogen for biochemical detection of native *Cg*-BigDefs. An experimental infection was further performed by injecting 5×10^7^ CFU/animal of live oyster pathogen *V. splendidus* LGP32 or live avirulent strain *V. tasmaniensis* LMG 20012^T^. Unchallenged oysters (i.e. oyster at time 0 h) and oysters injected with 100 µl SSW were used as controls. Hemolymph was withdrawn 3, 8, 24, 48, 72 and 120 h post-injection and hemocytes were collected and pooled (3 pools of 10 individuals per conditions) for RNA extraction. Additionally, unchallenged animals and oysters at 24 h post-injection with *V. splendidus* LGP32 were sampled for *in situ* hybridization. All experimental infections were performed according to the IFREMER animal care guideline and policy.

### Real-time quantitative PCR analysis

Total RNA extraction from oyster hemocytes and cDNA synthesis were performed as described above. Real-time quantitative PCR (qPCR) amplifications were performed in the LightCycler 480 (Roche) in a final volume of 6 µl containing 5 mM MgCl_2_, 0.5 µM of each primer, 3 µl of reaction mix (LightCycler 480 SYBR Green I Master 2X) and 1 µl of each reverse transcribed RNA (diluted 1∶19). Primer sequences are showed in **Table 2**. Each qPCR experiment was performed in triplicates and run under the following conditions: 95°C for 10 min; then 40 cycles of denaturation at 95°C for 10 s, annealing at 57°C for 20 s and extension at 72°C for 25 s. Results are presented as changes in relative expression normalized with the *C. gigas* ribosomal protein L40 reference gene (*Cg-rpl40*, GenBank: FP004478), using Pfaffl method [42]. Statistical significance was determined by Student's t-test at *p*<0.05.

### Analysis of *C. gigas* gill extracts by RP-HPLC and MALDI-TOF Mass Spectrometry

Native *Cg*-BigDefs were searched in gills from unchallenged and stimulated oysters (10 animals per conditions). Frozen gills were ground to fine powder. Samples were then diluted in 10% acetic acid, homogenized, and left at 4°C under gentle stirring for 15 h. Extracts were centrifuged at 8,000 ×g (20 min at 4°C), and further acidified with 0.05% trifluoroacetic acid (TFA). The RP-HPLC runs were carried out at 30°C on an Alliance system from Waters (Milford, MA, USA) coupled to a Photodiode array detector (Waters, Milford, USA). The separation was performed on an analytical C_18_ reversed-phase column (Vydac™, 218TP54, 4.6×250 mm, Protein – Peptide C_18_, 5 µm, Vydac Mojave, CA, USA). Extracts were purified using a linear gradient of 0–60% of acetonitrile in acidified water over 40 min at a flow rate of 0.6 ml/min. The column effluent was monitored for absorbance at 214 nm and the fractions were hand-collected following optical density, freeze-dried and conserved at −20°C until use. Finally, all collected fractions were analyzed by matrix-assisted laser desorption/ionization-time of flight (MALDI-TOF) mass spectrometry (MS) in a linear positive mode.

### *In situ* hybridization

Tissues from 24 h *Vibrio*-challenged and unchallenged oysters were prepared for histology and *in situ* hybridization (ISH) analyses as described by Muñoz *et al*. [43]. Specific primers (**Table 2**) were used to prepare cDNA probes for the analysis of *Cg*-BigDef1 and *Cg*-BigDef3 mRNA *in situ* expression. PCR products were cloned into a pCR®-Blunt II-TOPO® cloning vector that contains both SP6 and T7 promoters. The recombinant plasmids containing *Cg*-BigDef1 or *Cg*-BigDef3 cDNA (GenBank: JF703143 and JF703149, respectively) were then used as templates for the preparation of the probes. Digoxigenin (DIG)-UTP-labelled antisense and sense riboprobes were generated from linearized cDNA plasmids by *in vitro* transcription using RNA labelling kits, T7 and SP6 RNA polymerases (Roche). DIG-labelled riboprobes were hybridized on oyster tissue sections as described previously [43]. Control consisted in replacing antisense riboprobes with sense riboprobes.

## Supporting Information

Figure S1 **Nucleotide and deduced amino acid sequences (one letter code) of the three forms of big defensins from the oyster** ***Crassostrea gigas***: *Cg*-BigDef1 **(a)**, *Cg*-BigDef2 **(b)** and *Cg*-BigDef3 **(c)**. The predicted signal peptides are in bold and underlined. The putative propeptides are shadowed with grey background. Asterisks (*) mark the stop codon and the polyadenylation signals are double underlined.
(TIF)
