# Supplementary figures and images for: Big Defensins, a Diverse Family of Antimicrobial Peptides That Follows Different Patterns of Expression in Hemocytes of the Oyster Crassostrea gigas
Source: PLoS One. 2011 Sep 28;6(9):e25594. doi: 10.1371/journal.pone.0025594 (PMC3182236; doi:10.1371/journal.pone.0025594)

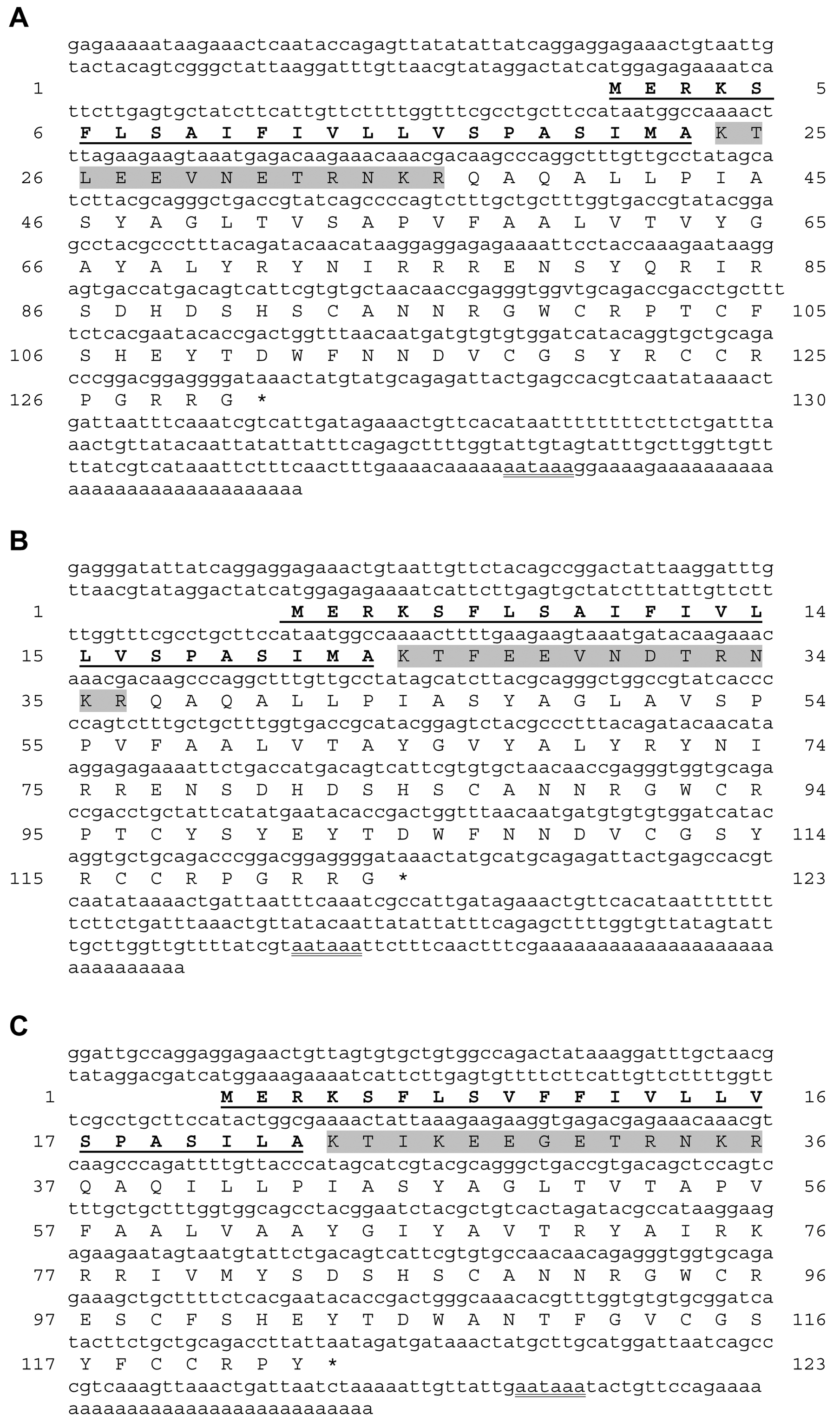

Supplement: Figure S1 — Nucleotide and deduced amino acid sequences (one letter code) of the three forms of big defensins from the oyster Crassostrea gigas: Cg-BigDef1 (a), Cg-BigDef2 (b) and Cg-BigDef3 (c). The predicted signal peptides are in bold and underlined. The putative propeptides are shadowed with grey background. Asterisks (*) mark the stop codon and the polyadenylation signals are double underlined. (TIF) [file pone.0025594.s001.tif]
